# Governance of tuberculosis control programme in Nigeria

**DOI:** 10.1186/s40249-019-0556-2

**Published:** 2019-06-17

**Authors:** Daniel Chukwuemeka Ogbuabor, Obinna Emmanuel Onwujekwe

**Affiliations:** 1 Department of Health Systems and Policy, Sustainable Impact Resource Agency, University of Nigeria Enugu Campus (UNEC), 22 Ogidi Street, Asata, Enugu, P.O. Box 15534, Enugu, Enugu State Nigeria; 2 Department of Health Administration and Management, University of Nigeria Enugu Campus, Enugu, Enugu State Nigeria; 3 Health Policy Research Group, College of Medicine, University of Nigeria Enugu Campus, Enugu, Enugu State Nigeria

**Keywords:** Nigeria, Tuberculosis, Control programme, Governance, Scoping review

## Abstract

**Background:**

The role of governance in strengthening tuberculosis (TB) control has received little research attention. This review provides evidence of how institutional designs and organisational practices influence implementation of the national TB control programme (NTP) in Nigeria.

**Main text:**

We conducted a scoping review using a five-stage framework to review published and grey literature in English, on implementation of Nigeria’s NTP and identified themes related to governance using a health system governance framework. We included articles, of all study designs and methods, which described or analysed the processes of implementing TB control based on relevance to the research question.

The review shows a dearth of studies which examined the role of governance in TB control in Nigeria. Although costed plans and policy coordination framework exist, public spending on TB control is low. While stakeholders’ involvement in TB control is increasing, institutional capacity is limited, especially in the private sector. TB-specific legislation is absent. Deployment and transfer of staff to the NTP are not transparent. Health workers are not transparent in communicating service entitlements to users. Despite existence of supportive policies, integration of TB control into the community and general health services have been weak. Willingness to pay for TB services is high, however, transaction cost and stigma among patients limit equity. Effectiveness and efficiency of the NTP was hindered by inadequate human resources, dilapidated service delivery infrastructure and weak drug supply system. Despite adhering to standardized recording and reporting format, regular monitoring and evaluation, revision of reporting formats, and electronic data management system, TB surveillance system was found to be weak. Delay in TB diagnosis and initiation of care, poor staff attitude to patients, lack of privacy, poor management of drug reactions and absence of infection control measures breach ethical standards for TB care.

**Conclusions:**

This scoping review of governance of TB control in Nigeria highlights two main issues. Governance for strengthening TB control programmes in low-resource, high TB burden settings like Nigeria, is imperative. Secondly, there is a need for empirical studies involving detailed analysis of different dimensions of governance of TB control.

**Electronic supplementary material:**

The online version of this article (10.1186/s40249-019-0556-2) contains supplementary material, which is available to authorized users.

## Multilingual abstracts

Please see Additional file 1 for translations of the abstract into the five official working languages of the United Nations.

## Background

Governance plays a key role in structuring responses to global health challenges such as tuberculosis (TB) but the role of governance in strengthening TB control has received little attention. Tuberculosis remains a major health problem in many low and middle-income countries (LMICs) including Nigeria, a country among the 30 high TB burden countries and one of the top three of ten countries that accounted for 80% of the total gap between TB incidence and reported cases in 2017 [1]. Only 20% of active TB cases in Nigeria are notified despite having the highest TB burden in Africa [2]. The TB prevalence rates in adults aged 15 years and above were estimated to be 318 per 100 000 population for smear-positive, and 524 for bacteriologically-confirmed cases in Nigeria [2]. In 2017, about 75% of the estimated 418 000 incident cases of TB in Nigeria were not notified or diagnosed and TB mortality (excluding Human Immunodeficiency Virus + TB) was 63 per 100 000 population [1]. Other factors associated with high TB burden in Nigeria are high proportion of patients with drug-resistant TB estimated at 4.3% among new cases and 25% among previously-treated cases [1]; and weak health systems that are unable to support efficient scale-up of TB services [3].

To eliminate TB as a major public health problem, Nigeria initially adopted directly observed treatment short-course (DOTS) strategy in 1993 [3]. DOTS strategy involved government commitment, case detection by sputum microscopy, short course treatment directly observed by a health worker; regular and uninterrupted drug supply; and standardised recording and reporting system. Implemented DOTS strategy led to improved sputum smear microscopy, rise in DOTS centres, increase in TB notification and attainment of the national TB treatment success target. The weakness in the DOTS strategy led Nigeria to adopt the Stop TB Partnership strategies which included DOTS expansion and enhancement; addressing tuberculosis-human immunodeficiency virus (TB-HIV), multi-drug resistant TB (MDR-TB) and the needs of the vulnerable populations; health systems strengthening for TB care; engaging all care providers; empowering people with TB and communities through partnership; and enabling and promoting research. In line with the sustainable development goals (SDGs), Nigeria embraced the End TB Strategy which emphasizes the central role of bold policies and supportive systems, universal health coverage (UHC) and health system governance in eliminating TB [3].

Governance encompasses a set of processes: institutions, rules, customs, policies or laws, that are formally or informally applied to distribute roles and responsibilities or accountability among societal actors [4]. Health system governance involves setting shared strategic direction and objectives for the health system; making policies, legislations, or decisions; raising and deploying resources to accomplish strategic goals and objectives; and ensuring that the strategic goals and objectives are accomplished [5, 6]. Governing the health system entails using strategic policy frameworks, effective oversight, coalition building, regulations and incentives, system design and accountability to achieve the goals of the health system [7]. In the National TB Control Programme (NTP), decisions must be evidence-informed, value-driven, transparent, inclusive, and responsive to the needs of the actors the TB programme serves; actors who make and those who implement decisions must be accountable; strategic objectives must be effectively, efficiently, ethically, and equitably met; and the vitality of the NTP and the services it provides must be sustained to attain good governance [8].

The national strategic plan for TB control (NSP-TB) prioritises health system governance to accomplish universal access to high quality, patient-centred prevention, diagnosis and treatment services for TB [3]. The NSP-TB aligns with the four components of governance within the national strategic health development plan (NSHDP). First, to provide clear policy directions for health development, the NSP-TB envisages joint strategic planning at federal-state and state-local levels to set strategic direction and objectives for TB control and raise and deploy resources to accomplish the strategic goals and objectives. The second is to facilitate legislation and a regulatory framework for health development, which involves advocacy for incorporation of TB considerations in all health policies and regulatory documents. The third is to strengthen accountability, transparency and responsiveness of the national health system. The NSP-TB envisions citizen oversight, annual reviews and external evaluations of the TB control programme. The fourth is to enhance the performance of the national health system, which involves rigorous monitoring and evaluation and continuous quality improvement processes in TB control interventions. Furthermore, the NSP-TB acknowledges that governance underlies service delivery, financing, human resources, information system, community participation, partnership and research for TB control.

Research evidence for TB control which focus primarily on how governance processes and practices inform the design and implementation of NTP interventions in LMICs are scarce. However, studies from settings outside Nigeria reveal poor governance practices. Absence of specific TB legislation, inconsistent enforcement of policies on isolation of TB patients in health facilities or incarceration [9, 10]; and weak regulatory framework for TB medicines [11] constrained the legal environment for TB control. Low government funding [12, 13]; inadequate human resources and lack of public awareness [14]; poor integration into the general health system [15]; weak programme implementation, sub-optimal quality of care in the private sector, and insufficient advocacy around TB [13] limited the strategic vision and responsiveness of TB control programmes. Weak institutional capacity of health facilities including stock-outs of drugs/supplies, inadequate spacing and infrastructure, lack of training, high workload, low staff motivation, and poor coordination of health centre services hindered efficient and effective TB service delivery [16]. High cost of TB care, despite free care policy, reduced equity in use of TB services [17–19]. Few studies highlight good governance practices. Foreign aid was effective in reducing incidence of TB [20]. Prioritisation of TB by decision makers, increased access to TB services in vulnerable population, greater participation of stakeholders from non-health sectors enhanced TB control [21, 22]. Integration of pharmacovigilance into TB control programme improved the management of adverse drug reactions [23]. Inclusion of TB in tax-funded health insurance schemes improved financial protection from use of TB services [24]. In India, citizens use public interest litigation to hold decision makers and providers accountable for rights violation and demand for new legislation, standards for TB patient care, public spending and quality of care [25].

There is a need to generate more evidence of the governance imperatives for achieving universal access to prevention, diagnosis and treatment of TB in resource-constrained countries. This paper aimed to answer the question what governance factors enable or constrain the implementation of TB control in Nigeria?, and to explore the significance of governance for TB control policy effectiveness will provide evidence that would be relevant to health system actors in Nigeria and other high TB burden countries in planning, design and implementation of TB care and prevention.

## Methods

We undertook a scoping review of grey and published literature on implementation of Nigeria’s TB control programme and identified themes related to health system governance. Scoping review was deemed an appropriate research design for this study because of the paucity of analytical reviews on the role of health system governance in TB control in Nigeria. Our review was based on the York methodology which included five stages namely, identifying the research question; identifying relevant studies; selecting the studies for review; charting the data; and collating, summarising and reporting results [26].

This review used the Siddiqi et al. health system governance framework [27]. We adapted the ten dimensions of governance — strategic vision, participation and consensus orientation, rule of law, transparency, responsiveness, equity and inclusiveness, effectiveness and efficiency, accountability, information and intelligence, and ethics – to explore the implementation of TB control programme. This framework was deemed appropriate because it permits ‘diagnosis of the ills’ in health system governance at policy and operational levels and identifies governance imperatives to improve health system performance [27].

In November 2018, we searched the main database in health and medicine, namely Scopus, MEDLINE, Complementary Index, Academic Search Complete, Science Citation Index, Directory of Open Access Journals, Supplementary Index, ScienceDirect and CINAHL for peer-reviewed articles published in English between 2000 and 2018. The search strategy was adapted for each database consistent with their indexing and used a combination of Mesh Terms or keywords with Boolean operators (and, or): tuberculosis, TB, control, programme, governance, management, implementation, evaluation, strategy, intervention, Nigeria and Nigerian. In addition, in consultation with the NTP officials, we identified policies and NTP reports to ensure comprehensive coverage of all sources providing information related to governance factors in TB control in Nigeria.

**Fig. 1:**
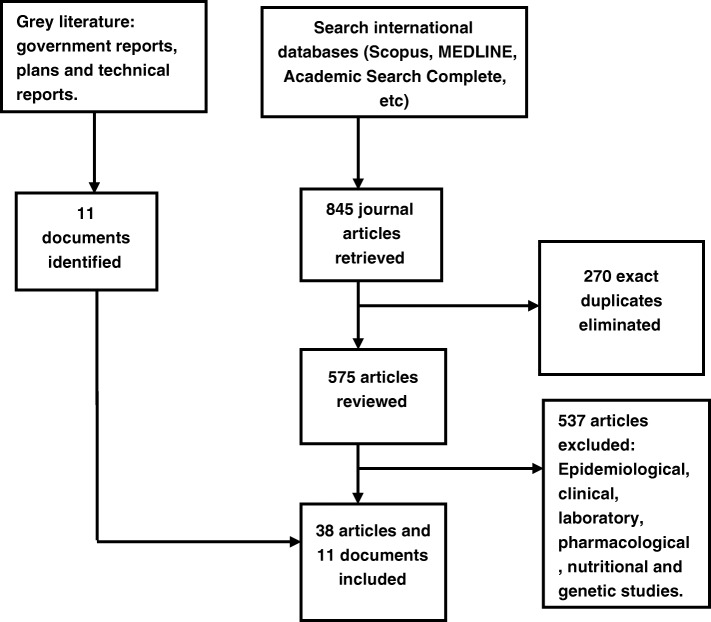
A flowchart showing the process of article selection for this review

Initially, we intended to include only studies focusing on governance analysis of the Nigeria’s TB control programme but found only one study directly related to governance of TB care on screening the titles of the studies. Following review of the abstracts and detailed examination of the studies, this review therefore included articles, of all study designs and methods, describing or analysing the processes of implementing TB control based on significance, level detail and relevance to the research question. The articles must focus on Nigeria, published in English language between 2000 to 2018 and findings included a governance outcome. No study was excluded based on risk of bias of the study. 270 articles were eliminated because they were exact duplicates, while 537 articles were excluded because they were one of epidemiological, clinical, laboratory, pharmacological, nutritional and genetic studies. The flowchart showing the process of article selection for this review is shown in Fig. 1. Overall, 38 journal articles and 11 documents which met the inclusion criteria were selected for review. Additional file 2 provides the characteristics of the studies included.

The selected documents were imported into NVivo software (version 11, QSR International Pty Ltd., Victoria, Australia). Codes were deduced from the ten dimensions of Siddiqi and colleagues’ health system governance framework (Table 1). We abstracted information that described governance processes and practices from the selected articles and documents and coded them to the appropriate dimensions of the governance framework.

**Table 1:** Applying Siddiqi et al. governance framework principles to tuberculosis (TB) control programme in Nigeria

Dimensions	Domains
*Strategic vision*	
Leaders should have strategic direction with clear priorities, roles and performance targets; and a shared long-term goal and strategic plan for health development	Enabling and constraining factors to development and implementation of plans and policies for TB control.
*Participation and consensus orientation*	
People should have voice in decision-making for health, either directly or through their legitimate intermediate institutions that represent their interests.	Enabling and constraining factors to coordination and consultation with service providers, service users and other sectors outside of TB programme and health.
*Rule of Law*	
Legal frameworks pertaining to health and standards, guidelines, policies, and regulations should be fair and enforced impartially.	Enabling and constraining factors to enforcement of public health laws and regulations governing TB control.
*Transparency*	
Processes, institutions and information needed to understand, and monitor health matters are directly accessible to relevant health system actors when and where they are needed.	Enabling and constraining factors to ensuring transparency in resource allocation, decision-making, appointment and transfer of staff in TB control programme.
*Responsiveness*	
Institutions and processes should try to serve all stakeholders to ensure that policies and programmes are responsive to health and non-health needs of its users	Enabling and constraining factors to integration of TB program into general health services as well as in the community, budget of TB and priority given to TB in resource allocation.
*Equity and inclusiveness*	
All men and women should have opportunities to improve or maintain their health and well-being.	Enabling and constraining factors to TB control equitable financing, access to services and anti-stigma programmes.
*Effectiveness and efficiency*	
Processes and institutions should produce results that meet population needs and influence health systems outcomes without waste of resources.	Enabling and constraining factors to ensuring human resources capacity building, infrastructural development and supply chain management of TB drugs and laboratory consumables.
*Accountability*	
Decision makers and service providers are answerable to the public and institutional stakeholders for processes and outcomes.	Enabling and constraining factors to enforcement of citizen-driven accountability in TB control programme.
*Intelligence and information*	
Timely generation, collection, analysis and dissemination of accurate information to provide evidence for informed decisions that influence behaviour of different actors and interest groups.	Enabling and constraining factors to implementation of electronic data management system, generation and use of data for wider system monitoring and decision-making in TB control.
*Ethics*	
Policies and institutional mechanisms should promote and enforce high ethical standards in healthcare and safeguard interests and rights of patients.	Enabling and constraining factors to enforcement ethical standards of care to ensure people-centred TB care.

Extracted data were collated, summarised and synthesised using a framework analysis approach, which allowed the extensive data from different studies and policy documents to be systematically organised and analysed. In each domain, enabling and constraining governance factors were collated and synthesised. Features of governance arrangements in a well-developed and performing health system in our reference framework guided the distinction between enabling and constraining governance factors.

## Results

Table 2 shows the enabling and constraining factors to the governance of TB control in Nigeria.

**Table 2:** Governance of national tuberculosis control programme (NTP) in Nigeria

Governance dimension	Constraints	Enablers
Strategic vision	Insufficient or delayed government funding	Existence of strategic plans for Tuberculosis (TB) Robust policy coordination framework
Participation and consensus orientation	Weak public-private mix for TB service delivery	Strong stakeholder involvement in policy development and service delivery
Rule of law	Weak legal regimen for isolation of TB patients	Assessment of legal environment for TB control completed.
	Absence of TB legislation and law regulating sale of anti-TB drugs	
Transparency	Absence of clear staff needs.	
	Frequent changes in leadership of NTP	
Responsiveness	Stigma by health workers and the public	Need-based drug distribution system
	Poor infrastructure	Policies support integration of TB into general health services and community.
	Weak linkage between TB and Maternal and child health services Weak collaboration between NTP and National Primary Health Care Development Agency. Lack of incentives for community volunteers	Use of community volunteers
Equity and inclusiveness	High transaction cost.	Free TB sputum microscopy and treatment policy
	Women, children and rural dwellers have poor access to TB care.	
	Exclusion of TB from national health insurance guidelines.	
Effectiveness and efficiency	Poor attraction of health workers to TB care	Existence of a national TB training school
	NTP lack authority to influence staff recruitment and distribution.	Well-structured laboratory network system. Introduction of new diagnostics
	Poorly motivated TB service providers	
	Poor service delivery infrastructure	
	Inadequate drug distribution from state store to health facilities.	
Accountability	Absence of formal social accountability initiatives	Strong civil society involvement
Intelligence and information	Incomplete and delayed quarterly reporting.	Adherence to World Health Organisation’s recording and reporting standard
	Poor storage of surveillance data	Regular and effective data review meetings
	Weak human resources capacity in data management	Frequent revision of reporting formats Transition from paper to an electronic data management system
	Inadequate coverage for childhood TB	Existence of national prevalence data
Ethics	Delays in TB diagnosis and initiation of care	Standards for TB care exist.
	Poor staff attitude Long waiting time Absence of TB infection control measures Prevalent informal payments	Existence of infection control guidelines

### Strategic vision

Strategic vision is enabled by existence of strategic plans for TB control and specific programme components, policy coordination framework and donor funding [3]. There is clarity of roles among the different tiers of government and partners but insufficient engagement coordination with states and local governments [3]. Constraints to strategic vision include insufficient or delayed government funding of TB by all tiers of government characterised by inadequate budgetary allocations and non-release of approved budget and counterpart funds [3, 28–31]. Government allocation to TB represents ‘*only a fraction of the budget needed for full implementation of the NSP’* [3]. In 2017, domestic and international funding of TB control were 8 and 16% respectively, result in 76% funding gap [1].

### Participation and consensus orientation

Relevant stakeholders are involved in the development and implementation of the strategic plan for TB and delivery of TB services [3]. Structures facilitating TB/HIV collaboration exist at all levels [3, 32], but integration is weak [28, 30]. Public-private partnership improved TB case detection and case holding [31, 33–36]. Existence of evidence-informed guidelines and a steering committee [28]; capacity building, stakeholders meeting and quarterly supervision [32] enabled engagement of the private sector in TB control. Besides constraints of inadequate training, poor staffing and weak infrastructure, it was also found that private sector providers do not comply with public health record keeping and patient monitoring requirements due to difficult-to-use recording and reporting system, lack of transparency in the management of TB patients and weak regulatory framework [28, 32, 35, 37].

### Rule of law

Several domestic legal and policy frameworks govern the prevention, testing, treatment and care of TB and establishes legal rights for people with TB and those vulnerable to the disease [37]. In spite of these, the legal environment for TB control is weak due to absence of TB-specific legislation, absence of laws regulating sale of anti-TB drugs, no clear policy on the isolation and involuntary isolation of people with TB that effectively balances the human rights of people with TB and protection of public health [37]; exclusion of TB from the National Vulnerable Group Health Insurance Fund; free health insurance to disadvantaged people in the proposed Bill for National Health Insurance Commission Act and National Health Insurance (NHI) operational guideline [3].

### Transparency

Two sub-themes emerged from the findings: lack of transparency in posting and transfer, and lack of transparency in entitlement to free TB care. Depolyment and transfer of staff is not transparent due to absence of clear staff needs, frequent changes in leadership of the NTP and NTP’s lack of authority to influence staff recruitment and distribution [3, 29]. Secondly, there is poor awareness of service entitlements and misinformation about TB among users. Consequently, health workers exploit users and ‘*even sell the supposedly free drug*’ [38]. Users have limited knowledge of, and were not aware of, the causative agent, the mode of transmission, or the designated clinics for effective diagnosis and management [28, 39–41]. Poor knowledge of TB was high among the poor, not educated, unemployed and rural residents [42].

### Responsiveness

NTP recognises the need to elicit the support and participation of community members and traditional healers in TB control efforts [3, 28, 43]. Community volunteers, family members and treatment support groups facilitate community TB care [38, 44, 45]. Community TB care is enabled by provision of incentives, appropriate selection of volunteers, supportive supervision, and a responsive TB programme [46]. Although policies support integration of TB into primary health care (PHC) facilities and the community [32], these policies are poorly implemented [28]. Integration into the PHC is limited by stigma by health workers, poor infrastructure, weak linkage between TB and maternal and child health services, and weak collaboration of NTP and national primary health care development agency (NPHCDA) [3, 28]. Factors limiting integration of TB control into communities include lack of clarity of roles of community-based organisations (CBOs) among stakeholders, lack of access to data for planning, weak technical and administrative capacity of CBOs, poor performance monitoring, factional CBOs and poor working relationships with NTP [3].

### Equity and inclusiveness

Notwithstanding the existence of a free TB sputum microscopy and treatment policy, TB patients pay hidden fees for services [3, 37]. Transaction costs and costs of additional investigations for the diagnosis of TB and co-morbidities are financial barriers of access to the diagnosis and treatment of TB in Nigeria [37, 47–51]. Women, children and rural dwellers have poor access to TB care [28]. Stigma is high, worse among women with TB and fuelled by misconceptions about TB [3, 28, 37, 45, 52–54]. However, the willingness to pay for TB services for own use and altruistic payments for the poor to have access to TB services are high [55]. Financial incentive was also found to improve TB treatment outcomes in rural Nigeria [56].

### Effectiveness and efficiency

Three sub-themes emerged — human resources, infrastructure and supply chain management. Human resources for adequate programme implementation are lacking in terms of the number of personnel, the requisite knowledge and skills and the level of motivation of staff across all levels of service delivery [3, 28]. There is no clear staff needs assessment [29]. A TB training school exits but attraction of health workers to TB care is poor due to stigma and safety concerns [29]. Performance of TB service providers is limited by poor salaries and working conditions, shortage of skilled TB workers, limited training opportunities and weak supervision [57] and poor adherence to NTP guidelines [58]. Whilst supervision is conducted on a regular basis, the content is superficial and follow-up is weak [3].

A well-structured laboratory network system [44] and new diagnostics have been introduced into the NTP [3], but the functioning is limited by poor infrastructure [32, 59], insufficient laboratory staff [28] and uneven distribution of diagnostic centres [3, 60]. Local governments are supposed to provide basic infrastructure and equipment for TB control through the publicly owned PHC facilities [3], but many of these centres are untidy and lack basic service delivery infrastructure [3, 61, 62]. As a result, majority of TB cases reported in Nigeria are notified by secondary level facilities [3].

Periodic assessment of the logistics system; development of a logistic management information system and introduction of e-TB manager enabled needs-based distribution of drugs and drug management in health facilities [3, 31, 32, 63–65]. However, the distribution systems for TB medicines from the state stores to the facilities and capacity of DOTS providers to manage commodities are inadequate [3, 32, 65]. Drug distribution is limited by lack of support for transportation, insufficient drug stores in health facilities and dilapidated zonal and state stores [3].

### Accountability

Social accountability in TB control is limited to involvement of civil society organisations in advocacy and awareness raising and development of advocacy, communication and social mobilization (ACSM) guidelines [32]. Although advocacy committees were established in states and local governments [28], citizens lack power in the governance of TB care due to absence of social accountability mechanisms like complaint box, service charter and health facility committees. The NSP-TB envisages establishment and/ or training of (existing) ward health committees to strengthen oversight of TB services in health facilities [3].

### Intelligence and information

Adherence to World Health Organisation’s recording and reporting standard, regular data review and statistical report, data- verification visits, annual joint missions, frequent revision of reporting formats, transition from paper to an electronic data management system, alignment of reporting formats to national health management information system, [3, 30, 44], and existence of national prevalence data [2] enabled information and intelligence. However, high proportion of missing records in health facilities [66], incomplete and delayed quarterly reporting [32, 44], poor storage of surveillance data, weak human resources capacity in data management, inadequate coverage for childhood TB and TB mortality surveillance [3, 28, 66], remain key constraints.

### Ethics

Although standards for TB care exist, ethics was breached by delays in TB diagnosis and initiation of care [67]; poor staff attitude to TB patients [38, 61, 68, 69]; long waiting time and poor service delivery infrastructure [61]; health system delays to TB diagnosis and treatment [70] lack of privacy involved in obtaining TB services [37]; significant variations in reporting adverse events from TB drugs [71] and absence of TB infection control measures including lack of infection control plan; infrastructure; lack of information, education and communication materials; no periodic screening of patients with cough or health workers; and nursing TB patients in the same ward with other vulnerable patients [28, 62, 72, 73].

## Discussion

This review shows that studies related to governing TB control in Nigeria are limited. Most studies describe or analyse the processes of implementing TB control, offering insights into how institutional designs and organisational practices affect the NTP in Nigeria. The application of the Siddiqi et al. governance framework to these studies and policy documents has revealed some enablers and many constraints to governing TB control in Nigeria.

The existence of strategic plans and guidelines for TB control, policy coordination framework, clarity of roles of different actors and donor funding facilitated TB control. Since inception of the NTP in 1991, several guidelines and strategic plans have been developed for TB control. The policies set clear priorities, define roles and performance targets as well as shared long-term goals for TB care and control. These findings align with WHO’s pillar 2 of the End TB Strategy, ‘bold policies and supportive systems’ for TB care and prevention. However, existence of policy coordination framework did not translate to effective coordination in practice. Although plans are costed, funding gaps persisted due to low public spending and unpredictable release of funds by all tiers of government. These findings are similar to the low government funding found in Pakistan and India [12, 13]. In our study, government budgetary allocations are insufficient and release of funds is often delayed or not effected at all. To improve the strategic vision for TB control, there is a need for effective policy coordination, increased budgetary allocations, and predictable release of funds to the NTP.

An increasing engagement of all stakeholders in the development and implementation of policies for TB control is another finding. The structures and guidelines for TB/HIV collaborative activities exist at both facility and programme management levels but programme implementation is weak. The NTP also recognises the need to engage the private sector using evidence-informed guidelines, capacity building, stakeholders’ consensus building meeting and supportive supervision. Nonetheless, the capacity of private health facilities to deliver TB services has been limited by poor organisational factors, inadequate human resources, non-compliance with patient monitoring requirements and weak regulatory framework, which results in sub-optimal quality of care as is the case in India [13]. Addressing these contextual factors would improve participation of the private sector in TB control.

A weak legal environment for TB control resulted from absence of TB-specific legislation, lack of laws regulating sale of anti-TB drugs, no clear policy on isolation and involuntary isolation of people with TB, and exclusion of TB from health insurance act. This finding is consistent with evidence from several studies highlighting absence of specific TB legislation, inconsistent enforcement of policies on isolation of TB patients in health facilities or incarceration [9, 10]; and weak regulatory framework for TB medicines [11]. A TB-specific legislation is needed to provide a legally binding governance mechanism for TB control in Nigeria that is consistent with a human rights-based approach. Such a law would mitigate emergence and spread of TB, balance the human rights of persons with TB and public health protection, accelerate expansion of universal coverage and social protection, and promote accountability [74].

Deployment and transfer of staff to the NTP is not transparent due to absence of clear staff needs, frequent changes in leadership of the NTP and NTP’s lack of authority to influence staff recruitment and distribution at all levels. Notwithstanding that staff allocation and training should be based on needs, primary health workers in Nigeria could be posted or transferred punitively, for political patronage or leniently closer to their residence [75]. Moreover, health workers are not transparent in communicating service entitlements and providing information to users, which limits their access to free TB care and patient-centred TB care. This finding is similar to low public awareness observed in Malaysia [14]. Transparency in TB control would improve when posting and transfer are based on needs assessment, and users of TB services are empowered with information that ensures unhindered access to TB care.

This review further indicates that integration of TB control into the community and general health services have been weak despite existence of supporting policies. Poor integration of TB into general health services was also seen in Vietnam [15]. Effective collaboration between the NTP and primary health care development agencies could enhance integration of TB into maternal and child health services, reduction of stigma among primary health workers, availability of TB service delivery infrastructure and community participation in TB care. Equally, appropriate selection and supervision of volunteers and provision of incentives using accredited community-based organisations would improve the responsiveness of TB programme.

Whereas transaction cost and stigma among patients limited equity and inclusiveness of TB care, the willingness to pay for TB services for self and others was high in this review. The findings about transaction cost is consistent with evidence of high cost of TB care despite free care policy in Burkina Faso, South Africa and China [17, 18, 76] and point to the need to include TB in universal health coverage schemes that offer financial protection [24]. The UHC schemes, which could also harness altruistic contributions, would address the need of the poor and vulnerable population and enhance sustainability of the TB programme [55, 76].

The finding that effectiveness and efficiency of the NTP was hindered by inadequate human resources, dilapidated service delivery infrastructure and weak drug supply system indicates the need for health systems strengthening for infectious diseases control. Similar to our findings, inadequate human resources for TB control was found in Malaysia and many TB high burden countries [14, 77]. Attraction and retention of health workers to the NTP would depend on stigma reduction among health workers, effective supervision and performance-linked incentives. Additionally, it is imperative to fill the gaps in service delivery infrastructure and supply of commodities in health facilities that offer TB care.

Citizen participation in TB control is low. Absence of social accountability initiatives limited citizens’ oversight of TB care despite the existence of advocacy, communication and social mobilisation policy. Conversely, greater participation of stakeholders was found in southeast Asian countries [21], and in India, citizens use public interest litigation to hold health decision makers and providers accountable for TB control [25]. It is imperative that decision makers and service providers in Nigeria’s NTP are responsive and accountable to the citizens [8] . Health facility committees (HFCs) exist in Nigeria and could serve as an entry point for advancing the standards for TB care, complaints system, participation in decision making and oversight of TB care. Hence, TB control should be emphasised in the operational guidelines of HFCs.

We also found that adherence to standardized recording and reporting format, TB prevalence survey, regular monitoring and evaluation, revision of reporting formats, electronic data management system and training of health workers are key factors that could strengthen TB surveillance system in Nigeria. Yet, TB surveillance system in Nigeria is weak similar to experiences in South Africa [78]. Mathematical models have been advocated to be incorporated into surveillance systems [79], but there is yet no application to TB surveillance in Nigeria. It might be helpful to improve the integrity of the surveillance system and its linkage to evidence-informed TB policy and practice.

The review findings also highlight the need for adherence to ethical standards for TB care. Delay in TB diagnosis and initiation of care resulting in long waiting times, diagnostic drop outs and loss before treatment; poor staff attitude to patients; lack of privacy; poor management of drug reactions and absence of infection control measures, confirm the breach of both the international standards for TB care and patient-centred TB care [80, 81]. Since patient-centred TB care is associated with better treatment adherence, improved patient outcome and quality of life of patients with TB [82], the NTP must address diagnostic and treatment delays, patients’ needs and expectations, adverse drug events and TB infection control in health facilities.

## Conclusions

This study fills a significant gap in knowledge about the role and importance of governance in TB control in Nigeria. It addressed this gap by applying the governance framework of Siddiq and colleagues to identify factors that either enabled or constrained effective implementation of Nigeria’s NTP. Secondly, it contributes to the policy debate on health system strengthening for infectious disease control programmes. Evidence from the review can be used by LMICs to improve the design and implementation of their TB control programmes. This study is, however, limited by paucity of studies on many dimensions of governance. Further empirical studies, involving detailed analysis of the different aspects of governance, are needed.

In conclusion, this review has identified the governance imperatives to strengthen TB control programme in Nigeria and similar settings. Effective governance for TB control would entail adequate policy coordination; increased, predictable TB financing; and collaborative design and implementation of strategies for TB control. Stronger legal and regulatory environment consistent with a human rights-based approach would be helpful. Transparent posting and transfer of staff; clear communication of service entitlements and obligations to patients are imperative. Equally, need-based integration of TB control into communities and general health services; and inclusion of TB services in UHC schemes are warranted. It is also necessary to ensure effective organisation of service delivery; adequate and motivated health workforce; regular and uninterrupted drug supply; and stronger roles for citizens in policy engagement and oversight of health facilities. Furthermore, functional TB surveillance system, and adherence to ethical standards for TB care are crucial.

## Additional files

Additional file 1: Multilingual abstracts in the five official working languages of the United Nations. (PDF 567 kb)

Additional file 2: Descriptive content of the 38 selected articles and 11 documents reviewed. The file provides the detailed characteristics of the journal articles and documents included in this scoping review. The table includes authors, year of publication, methods, population and sample, key outcome and conclusion. (DOCX 32 kb)

## Data Availability

Data sharing is not applicable to this article as no datasets were generated or analysed during the current study.

## Abbreviations

ACSM: Advocacy, communication and social mobilisation
CBOs: Community based organisations
DOTS: Directly observed treatment short-course
e-TB: Electronic-tuberculosis
HFC: Health facility committee
LMICs: Low- and middle-income countries
MDR-TB: Multi-drug resistant tuberculosis
NHI: National health insurance
NPHCDA: National primary health care development agency
NSHDP: National strategic health development plan
NSP-TB: National strategic plan for tuberculosis control
NTP: National TB control programme
SDGs: Sustainable development goals
TB: Tuberculosis
TB/HIV: Tuberculosis/ Human immunodeficiency virus
UHC: Universal health coverage
WHO: World Health Organisation
