# Supplementary material for: Governance of tuberculosis control programme in Nigeria
Source: Infect Dis Poverty. 2019 Jun 17;8:45. doi: 10.1186/s40249-019-0556-2 (PMC6572737; doi:10.1186/s40249-019-0556-2)
Supplement: Supplementary file 1 — Multilingual abstracts in the five official working languages of the United Nations. (PDF 567 kb) [file 40249_2019_556_MOESM1_ESM.pdf]

Translation of the abstract into the five official working languages of the United Nations

**إدارة برنامج مكافحة السُل في نيجيريا**

*دانيال تشوكويميكا أوغبور وأوبينا إيمانويل أونوجوي*

**نبذة مختصرة**

**الخلفية**: حظي دور الإدارة في تعزيز مكافحة مرض السُل باهتمام بحثي ضئيل. تُقدم هذه المراجعة دليلاً على طريقة تأثير التصميمات المؤسسية والفعاليات التنظيمية على تطبيق البرنامج المحلي لمكافحة مرض السُل في نيجيريا.

**النص الرئيسي**: أجرينا استعراضاً استطلاعياً باستخدام إطار مكون من خمس مراحل؛ لمراجعة أبحاث غير رسمية منشورة باللغة الانجليزية، حول تنفيذ بروتوكول وقت الشبكة (NTP) في نيجيريا وتطبيق موضوعات محددة متعلقة بالإدارة باستخدام إطار إداري ذو نظام صحي، وأدرجنا مقالات بكل التصميمات والأساليب التي تصف أو تحلل عمليات تنفيذ مكافحة السُل بناءاً على علاقتهم بسؤال البحث.

تُظهِر المراجعة نُدرة في الدراسات التى تدرس دور الإدارة في مكافحة مرض السُل في نيجيريا، على الرغم من وجود خطط مُكلِفَة وإطار من السياسات المُنسقة، فإن الميزانية العامة على مكافحة مرض السُل منخفضة، وفي حين أن مشاركة أصحاب المصالح لمكافحة مرض السُل في تزايد، القُدرة المؤسسية محدودة، خصوصا في القطاع الخاص؛ فالتشريعات الخاصة بمرض السُل غائبة. انتشار ونقل الموظفون إلى بروتوكول وقت الشبكة ليس واضحاً. الموظفون الأصحاء ليسوا شفافين في توصيل استحقاقات الخدمة للمستخدمين، وعلى الرغم من وجود سياسات داعمة، ضَعُفَ دمج مكافحة مرض السُل في المجتمع وخدمات الصحة العامة. إن الرغبة في الدفع مقابل خدمات مرض السُل مرتفعة، ومع ذلك، تكلفة المعاملات والتمييز بين المرضى تَحُد من الإنصاف. فعالية وجدارة بروتوكول وقت الشبكة مُنعت من قبل موارد بشرية غير مؤهلة، وبنية تحتية متداعية لتقديم الخدمات، ونظام ضعيف للتمديد بالعقاقير. وعلى الرغم من الالتزام بتسجيل وتصميم تقارير موحد، ومراقبة عادية وتقييم، ومراجعة لتصاميم التقارير، ونظام إدارة البيانات الالكترونية، فإن نظام مراقب السُل وُجِدَ ليكون ضعيفاً. التأخير فى تشخيص مرض السُل والبدء في الرعاية، وسوء سلوك الموظفين تجاه المرضى، وانعدام الخصوصية، والإدارة السيئة للتفاعلات الدوائية، وعدم وجود تدابير لمكافحة العدوى ينتهك المعايير الأخلاقية للعناية بمرضى السُل.

**الاستنتاجات**: هذا الاستعراض الاستطلاعي للإدارة لمكافحة مرض السُل في نيجيريا يُسلط الضوء على مسألتين رئيسيتين. حوكمة تعزيز برامج مكافحة مرض السُل في البيئات المنخفضة الموارد والعالية العبء للسل مثل نيجيريا، هو أمر حتمي. ثانيا، هناك حاجة للدراسات التجريبية تتضمن تحليلاً مفصلاً للأبعاد المختلفة لإدارة مكافحة مرض السل.

Translated from English version into Arabic Abdallah Hamdy, Revised by Sondos Hany, through

TRANSLATORS WITHOUT BORDERS

## 尼日利亚结核病控制项目的监管

Daniel Chukwuemeka Ogbuabor and Obinna Emmanuel Onwujekwe

**摘要**

**引言：**研究者很少关注监管在加强结核病控制方面的作用。本综述论证了尼日利亚的机构设置和政府行为如何影响国家结核病控制规划（NTP）的实施。

**正文：**我们采用五步框架法审阅了已发表的和灰色文献，使用卫生系统治理框架确定了与监管相关的主题，对尼日利亚国家结核控制规划的实施情况开展了辖域综述。在所有研究设计和方法中，我们囊括了一些与研究问题相关或分析结核病控制实施过程的文章。

本文发现，少有研究关注尼日利亚结核病控制中监管作用。虽然已有成本计划和政策协调框架，但结核病控制方面的公共支出依然很低。尽管越来越多的利益攸关方参与结核病控制，但机构尤其是私营部门能力有限。特定结核病立法缺失。国家结核控制规划的人员部署和流动不透明，向患者提供服务的卫生工作者也不固定。虽然有支持性政策，但难以将结核病控制纳入社区和一般卫生服务工作。支付结核病服务的意愿很高，但是，治疗成本和歧视限制了医疗公平。受人力资源不足、基础服务设施破旧和药物供应系统薄弱的阻碍，国家结核控制规划的效力和效率均较低。虽然结核病监测体系坚持规范的记录和报告格式，定期进行监测和评价、修订报告格式，并建立电子病历管理系统，但其仍存在薄弱环节。还有一些其他行为违反了结核病治疗的伦理标准，如结核病诊断和治疗的延误，工作人员对患者态度差，缺乏隐私管理，药物反应管理不佳以及缺乏感染控制措施。

**结论:**尼日利亚结核病控制监管存在两个突出的问题。首先在尼日利亚这样资源少、结核病负担高的国家，加强结核病控制规划的监管势在必行。其次，亟需开展不同层面结核病控制监管的实证研究。

Translated from English version into Chinese by Peng Song, edited by Jin Chen, through

CHINESE CENTER FOR DISEASE CONTROL AND PREVENTION
NATIONAL INSTITUTE OF PARASITIC DISEASES

## Gouvernance du programme de lutte contre la tuberculose au Nigéria

Daniel Chukwuemeka Ogbuabor and Obinna Emmanuel Onwujekwe

**Résumé**

**Contexte :** Le rôle de la gouvernance dans le renforcement du contrôle de la tuberculose a fait l'objet de peu de recherches. Cet examen fournit des preuves de la manière dont les conceptions institutionnelles et les pratiques organisationnelles influencent la mise en œuvre du programme national de contrôle de la tuberculose au Nigeria.

**Texte principal :** Nous avons mené une étude exploratoire grâce à un plan en cinq étapes afin d'examiner la documentation publiée et parallèle en anglais sur la mise en œuvre du programme nigérian de contrôle de la tuberculose. Nous avons également identifié les thèmes en rapport avec la gouvernance grâce à un cadre de gouvernance du système de santé. Nous avons inclus des articles comprenant tous les modes et plans d'étude qui ont décrit ou analysé les modalités de mise en œuvre du contrôle de la tuberculose, selon leur rapport avec le sujet de recherche.

L'analyse révèle que peu d'études ont été faites sur le rôle de la gouvernance dans le contrôle de la tuberculose au Nigéria. Bien que les plans chiffrés et les systèmes de coordination des politiques existent, les dépenses publiques engagées dans le contrôle de la tuberculose sont faibles. Même si

la participation des parties prenantes dans le contrôle de la tuberculose est en augmentation, la capacité institutionnelle est limitée, notamment dans le secteur privé. Il n'existe aucune loi liée à la tuberculose. L'affectation et le transfert du personnel dans le programme de contrôle de la tuberculose n'est pas transparent. Les professionnels de santé ne communiquent pas clairement aux usagers les prestations auxquelles ils ont droit. Malgré l'existence de politiques de soutien, l'intégration du contrôle de la tuberculose dans la communauté et les services généraux de santé sont faibles. Nombre sont ceux qui sont prêts à payer pour bénéficier des services pour la tuberculose, mais les frais de transaction et la honte ressentie par les patients limitent les fonds propres. L'efficacité et l'efficience du programme de contrôle de la tuberculose ont été entravées par des ressources humaines inadaptées, une infrastructure de prestations de services décrépite et un système d'approvisionnement en médicaments insuffisant. Même en adhérant aux normes de modèle de présentation des enregistrements et des rapports, d'évaluation et de contrôles réguliers, de révision des modèles de présentation des rapports, et de système de gestion des données électroniques, le système de surveillance de la tuberculose s'est révélé être fragile. Les retards dans les diagnostics de la tuberculose et les prises en charge, l'attitude laissant à désirer du personnel à l'égard des patients, le manque d'intimité, la mauvaise gestion des réactions aux médicaments et l'absence de mesures de contrôle des infections constituent une violation des normes éthiques de prise en charge de la tuberculose.

**Conclusions :** Cette étude exploratoire de la gouvernance du contrôle de la tuberculose au Nigéria met en avant deux problèmes. Une gouvernance destinée à renforcer les programmes de contrôle de la tuberculose dans les milieux à faibles ressources et connaissant un nombre important de cas de tuberculose, comme le Nigéria, est impérative. Ensuite, il est nécessaire de conduire des études empiriques comportant des analyses détaillées des différents aspects de la gouvernance du contrôle de la tuberculose.

Translated from English version into French by Emilie Vandapuye, Revised by Ahmad Dabaghzadeh, through

TRANSLATORS
WITHOUT BORDERS

**Управление программой по борьбе с туберкулезом в Нигерии**

Daniel Chukwuemeka Ogbuabor and Obinna Emmanuel Onwujekwe

**Резюме**

**Справочная информация:** вопрос роли управления программой в усилении борьбы с туберкулезом (ТБ) не получал должного внимания. В этой рецензии представлены данные о влиянии институциональных моделей и организационных практик на внедрение национальной программы по борьбе с туберкулезом (НПБ ТБ) в Нигерии.

**Основная часть.** Мы провели предварительный анализ с использованием пятиступенчатой рамочной программы для поиска по рецензируемой и внеиздательской литературе на английском языке по вопросу внедрения НПБ ТБ в Нигерии и определили темы, связанные с управлением, используя рамочную программу по управлению системой здравоохранения. Мы включили статьи со всеми исследовательскими моделями и методами, в которых приводилось описание или анализ процессов осуществления борьбы с туберкулезом (ТБ) с учетом актуальности исследуемого вопроса.

В ходе обзора было выявлено наличие недостаточного количества исследований, в которых бы изучалась роль управления программой по борьбе с туберкулезом (ТБ) в Нигерии. Несмотря на наличие планов с полным расчетом расходов и рамочной программы по координации политики, государственные расходы на борьбу с ТБ остаются низкими. Наблюдается все более широкое участие заинтересованных сторон, но институциональные возможности ограничены, в частности частный сектор. Отсутствие конкретного законодательства по принципам борьбы с ТБ Распределение и перевод сотрудников в рамках НПБ не являются прозрачными. Работники здравоохранения не являются открытыми при передаче служебных прав пользователям. Несмотря на существование вспомогательных политик, интеграция борьбы с ТБ в общество и предоставление общих медицинских услуг остаются на низком уровне. Желание получать платные услуги по диагностике и лечению ТБ высоко. Однако вследствие операционных издержек и отрицательного отношения к пациентам условия для равноправного доступа ограничены. Достижение эффективности и результативности НПБ сдерживается нехваткой людских ресурсов, наличием полуразрушенной инфраструктуры предоставления услуг и низким уровнем системы поставки лекарственных препаратов. Несмотря на соблюдение стандартной формы для представления сведений и формата представления отчетности, проведения регулярного мониторинга и оценки, обзора форматов представления отчетности и электронной системы управления данными система наблюдения за ТБ остается слабой. Несвоевременная постановка диагноза ТБ и начало лечения, плохое отношение персонала к пациентам, отсутствие конфиденциальности, недостаточное наблюдение за реакциями на лекарственные препараты и отсутствие мер по контролю за инфекцией являются нарушением этических стандартов лечения ТБ.
<**Выводы:** В данном предварительном обзоре управления программой по борьбе с ТБ в Нигерии рассматриваются две основные проблемы. Управление программой для усиления борьбы с ТБ в условиях нехватки ресурсов и с тяжелым бременем ТБ, как в Нигерии, является неотложной задачей. Далее существует необходимость проведения эмпирических исследований, включая подробный анализ различных аспектов управления программой по борьбе с ТБ.

Translated from English version into Russian by Veronika Demeshchik, Revised by Alexander Somin, through

TRANSLATORS WITHOUT BORDERS

## La gobernanza del programa de control de la tuberculosis en Nigeria

Daniel Chukwuemeka Ogbuabor y Obinna Emmanuel Onwujekwe

**Resumen**
**Antecedentes:** el papel de la gobernanza en el fortalecimiento del control de la tuberculosis (TB) no ha sido muy investigado. Este estudio presenta pruebas de cómo los diseños institucionales y las prácticas organizativas influyen en la implementación del programa nacional de control de la tuberculosis (PNT) en Nigeria.
**Texto principal:** realizamos una revisión del alcance usando un marco de cinco etapas para revisar documentos publicados y literatura gris en inglés sobre la implementación del PNT en Nigeria, e

identificamos temas relacionados a la gobernanza usando un marco de gobernanza del sistema de salud. Incluimos artículos, de todos los diseños y métodos de estudio, que describían o analizaban los procesos de implementación del control de la TB sobre la base de la relevancia para la pregunta de investigación.

Esta revisión muestra la escasez de estudios que examinan el papel de la gobernanza en el control de la TB en Nigeria. A pesar de que existen planes con costes calculados y un marco de coordinación de políticas, el gasto público en el control de la TB es bajo. Si bien la participación de las partes interesadas en el control de la TB está aumentando, la capacidad institucional es limitada, especialmente en el sector privado. Falta una legislación específica sobre la TB. El despliegue y el traslado de personal para el PNT no son transparentes. Los trabajadores sanitarios no son transparentes a la hora de comunicar a los usuarios los derechos a los servicios. A pesar de que existen políticas de apoyo, la integración del control de la TB en los servicios de salud comunitarios y generales ha sido escasa. La disposición a pagar por servicios relacionados con la TB es elevada; sin embargo, los costes de transacción y el estigma entre los pacientes limitan la equidad. La efectividad y la eficiencia del PNT se vieron entorpecidas por la insuficiencia de recursos humanos, el deterioro de la infraestructura para la prestación de servicios y un sistema de abastecimiento de medicamentos débil. A pesar de la adhesión al registro estandarizado y el formato de informes, la evaluación y supervisión constantes, la revisión de los formatos de informes y el sistema electrónico de gestión de información, se observa que el sistema de vigilancia de la TB es débil. El retraso en el diagnóstico de la TB y en el inicio del tratamiento, la mala actitud del personal hacia los pacientes, la falta de privacidad, la mala gestión de las reacciones a los fármacos y la falta de medidas de control de la infección incumplen los estándares éticos para el cuidado de la TB.

**Conclusiones:** esta revisión del alcance de la gobernanza del control de la TB en Nigeria destaca dos problemas principales. Es imperativa la gobernanza para fortalecer los programas de control de la TB en entornos de bajos recursos y alta incidencia de TB, como Nigeria. Además, se necesitan estudios empíricos que involucren análisis minuciosos de las diferentes dimensiones de la gobernanza del control de la TB.

Translated from English version into Spanish by Abdallah Hamdy, Revised by Sondos Hany, through

TRANSLATORS
WITHOUT BORDERS
