# Supplementary material for: Governance of tuberculosis control programme in Nigeria
Source: Infect Dis Poverty. 2019 Jun 17;8:45. doi: 10.1186/s40249-019-0556-2 (PMC6572737; doi:10.1186/s40249-019-0556-2)
Supplement: Supplementary file 2 — Descriptive content of the 38 selected articles and 11 documents reviewed. The file provides the detailed characteristics of the journal articles and documents included in this scoping review. The table includes authors, year of publication, methods, population and sample, key outcome and conclusion. (DOCX 32 kb) [file 40249_2019_556_MOESM2_ESM.docx]

| **Additional file 1 Descriptive content of the 38 selected articles and 11 documents reviewed** | | | | | |
| --- | --- | --- | --- | --- | --- |
| **Authors[reference]** | **Date of publication** | **Method** | **Population and sample** | **Main outcome** | **Conclusion** |
| Otu AA. [28] | 2013 | Literature review | Annual programme reports and peer reviewed articles | Weaknesses include inadequate funding; weak TB/HIV integration is weak; weak public-private mix (PPM) for TB service delivery; poor integration of TB into PHCs and community; gender and rural inequities in access to TB care; poor infection control; and health workforce crisis. | Addressing weaknesses in the health systems will enhance reduction of the burden of TB in Nigeria |
| Awofeso N, Schelokova I, Dalhatu A.[29] | 2008 | Literature review | Published and grey literature | Insufficient budget for TB control in low resource countries mainly used to pay salaries of health workers | Appropriate (re)training of front-line health workers is a necessary but not sufficient activity for improving health worker performance as well as the quality of tuberculosis control outcomes. |
| Adejumo O, Olusoji D, Otesanya A, Salisu-Olatunji S, Abdul-Razzaq H. [33] | 2017 | Comparative study design based on quantitative review of treatment records. | Eleven of 31 private TB treatment centres in Lagos State that have microscopy centers and had been involved in NTP for at least 2 years. | The odds that patients treated at private-for-profit facilities had treatment success were about four times higher than private non-for-profit TB facilities. | Need to engage more private practitioners to increase case detection and improve treatment outcomes of TB patients. |
| Asuquo AE, Pokam BDT, Ibeneme E, Ekpereonne E, Obot V, Asuquo PN. [34] | 2015 | Cross-sectional study on Knowledge, attitude and practices (KAP). | The study was carried out in 18 communities in 6 selected local government areas (LGAs) of Akwa Ibom State. | Although knowledge of TB and its treatment is good, care-seeking attitude was poor due to stigmatization. Public-private partnership facilitated active TB case finding. About 98% of TB cases detected were linked to NTP. | Active case finding through public–private partnership can reduce TB burden. |
| Gidado M, Ejembi CL. [35] | 2009 | A comparative cross-sectional descriptive study | Five private and 10 public health facilities providing TB services for at least two years in four Local Governments Areas in Kaduna State, 15 facility heads and 492 case notes. | Public-private mix (PPM) for TB improved case detection and case holding; non-compliance with public health record keeping and patient monitoring requirement by private providers | Private health facilities which adhered to national guidelines had higher TB patient case load and better treatment outcome than public health facilities in Kaduna State |
| Onyeneho NG, Chukwu JN. [36] | 2010 | Mixed methods study - structured questionnaire, in-depth interviews and focus-group discussions (FGDs). | 388 patent medicine vendors (PMVs), 17 principal officers of PMV associations, and 17 community leaders were purposively selected. | Most (90%) PMVs indicated that they would be ready to cooperate with the NTP | The PMVs are widely accepted in the study area and positively disposed to playing roles in TB control. |
| Okeibunor JC, Onyeneho GC, Chukwu JN, Post E.  [38] | 2007 | Qualitative study using in-depth interview (IDIs) and focus group discussions (FGDs). | Community leaders, health workers, patent medicine vendors (IDIs) and community members (FGDs). | Barriers to TB care include users’ awareness about TB, availability and perceived efficacy of TB treatment services; perceived high cost of TB services; informal payment, and hostile attitude of providers. | The attitude of health workers must be addressed if community members will use TB treatment centres. |
| Enwuru CA, Idigbe EO, Ezeobi NV, Otegbeye AF. [39] | 2002 | Cross-sectional study to assess the effects of KAP on care-seeking behaviour. | Two chest referral clinics in Lagos, Nigeria, between February 2000 and May 2001; 168 newly diagnosed tuberculosis (TB) patients | Overall there was a low level of knowledge and awareness of the disease as well as an apparently high level of improper health care-seeking behaviour | Need to educate communities and care providers on the cause and mode of transmission of TB, and the need to attend designated health facilities for early diagnosis and proper treatment |
| Anochie PI, Onyeneke EC, Onyeozirila AC, Igbolekwu LC, Onyeneke BC, Ogu AC. [40] | 2013 | Mixed methods: Cross-sectional questionnaire survey; IDIs and FGDs | Multi-stage sampling was used to select 1186 people from a rural community in Aboh Mbaise LGA of Imo State. | Most people are aware of TB but have poor knowledge of cause of TB. Few people TB is curable with anti-TB medication. Stigma is high. | The evidence should inform community mobilization interventions for TB control. |
| Tobin E, Okojie P, Isah E. [41] | 2013 | Cross-sectional study to assess KAP regarding TB and its treatment. | 193 persons was carried out in a rural community in Ward 5 of Etsako-West local government area of Edo state, selected through a multi-stage sampling process | Majority of respondents had heard of TB, but their knowledge of the etiology, mode of transmission, and manifestations of TB are poor with the educated generally having a better knowledge of TB. Attitude toward TB was poor. | Knowledge and attitude toward TB were generally poor in this rural community |
| Agho KE, Ewald B. [42] | 2014 | Secondary analysis of NDHS data | Nationally-representative sample consisting of 34,644 households (were selected by 2-stage sampling) of which 34,070 were successfully interviewed. | Poor knowledge of TB high among the poor, uneducated, unemployed and rural residents | Efforts to improve the knowledge of and attitude towards TB among the youngest age-group (15-19 years), the poorest households, and persons with no schooling |
| Onyeneho NG, Chukwu JN. [43] | 2011 | Qualitative study based in-depth interviews | Community leaders (n=17) and Traditional healers (n=20) purposively selected from 9 communities in 6 LGAs in 3 states from southern Nigeria. | Participation of traditional healers in TB control | Traditional Healers can be educated to make prompt referral of cases to NTP |
| Balogun M, Sekoni A, Meloni ST, Odukoya O, Onajole A, Longe-Peters O, et al. [45] | 2015 | Cross-sectional survey, comparative (before and after) study. | 10 community volunteers from one LGA of Lagos State. | Community volunteers facilitate community TB care. | Trained community volunteers improved TB knowledge and attitudes of community members. |
| Adejumo AO, Azuogu B, Okorie O, Lawal OM, Onazi OJ, Gidado M, et al. [46] | 2016 | Comparative case study, mixed methods: quantitative document review and in-depth interviews. | Community workers, LG TB officer, CBOs and implementing partners in 3 Nigerian States of Lagos, Abia and Oyo. | Four models of referral compared. The annual mean referral per community worker is 13.4. The model with irregularly supervised, trained, and compensated CWs contributed the least to TB case detection. | Provision of incentives, appropriate selection of volunteers, supportive supervision, and a responsive TB programme enable community TB Care |
| Abimbola S, Ukwaja KN, Onyedum CC, Negin J, Jan S, Martiniuk ALC. [47] | 2015 | Cross-sectional survey | 452 TB patients from 3 secondary health centres for TB in Ebonyi State. | 84% of TB patients first consulted an inappropriate provider; 62% of transaction costs were incurred during the first visit to an inappropriate provider. The mean transaction costs incurred was highest with qualified providers. | Effective decentralisation, community engagement, enforcing regulation and referral linkage may reduce transaction costs. |
| Ukwaja KN, Alobu I, Igwenyi C, Hopewell PC. [48] | 2013 | Cross-sectional cost of illness study | 452 TB patients from 3 secondary health centres for TB in Ebonyi State. | Most patients borrow money, sell assets, or both to cope with the cost of care. Following an episode of TB, household income reduced increasing the proportion of households classified as poor from 54% to 79%. | Patient and household costs for TB care are potentially catastrophic even where services are provided free-of-charge. |
| Ukwaja KN, Alobu I, Igwenyi C, Hopewell PC. [49] | 2013 | Cross-sectional cost of illness study | 452 TB patients from 3 secondary health centres for TB in Ebonyi State. | Average direct household costs for TB were 14% of average annual incomes. The incidence catastrophic payment was 44%; with 69% and 15% of the poorest and richest household income-quartiles experiencing catastrophic activity. | Households incur catastrophic out-of-pocket payments for TB care. Financial and social protection interventions are needed for identified at-risk groups. |
| Umar N, Fordham R, Abubakar I, Bachmann M. [50] | 2012 | A cross sectional survey | 242 TB patients were sampled from 27 out of 67 facilities providing TB services in Bauchi state of Nigeria | Variable income and productivity loss among TB patients and households. Hospitalization and facility of diagnosis predicted income and productivity losses. | Tuberculosis poses tremendous burden in terms of time and productivity lost to both patients and their households in Bauchi State Nigeria |
| Umar NA, Abubakar I, Fordham R, Bachmann M. [51] | 2012 | Cost of illness study - cross sectional survey and quantitative document review | Officers in charge of 27 health facilities (17 PHCs, 9 SHCS and 1 THC) in Bauchi State. | The overall cost and all computed cost elements, except for DOT services, were highest in the tertiary centre and least expensive in the infectious diseases hospital. | The costs of anti-tuberculosis treatment are expensive and potentially catastrophic for many patients and their families |
| Oluwadare C, Bosede I. [52] | 2017 | Mixed methods: cross-sectional questionnaire survey and in-depth interview. | 117 TB patients were selected from the chest clinic register of the State Specialist Hospital and at family level using snowball method. | Perceived cost of treatment and fear of stigma limit access to TB services of HIV positive persons. | Improved TB treatment serves as veritable entry point for HIV screening and treatment |
| Abioye IA, Omotayo MO, Alakija W. [53] | 2011 | cross-sectional study, self-administered questionnaire | 205 patients on treatment at two government-owned referral centres for tuberculosis in Lagos State. | Eighteen percent reported a previous stigma experience. Stigma experience was significantly predicted by age, low socio-economic status, education below secondary level, disclosure of status, history of weight loss, previous smoking and alcohol history | Experience of stigma among patients with tuberculosis is common and may adversely affect treatment adherence. |
| Oshi DC, Oshi SN, Alobu IN, Ukwaja KN. [54] | 2016 | Qualitative study (In-depth interviews) | Maximum variation sampling of 56 participants (46 women and 10 men) from six communities in 3LGAs (one from each of 3 zones) of Ebonyi state. | Stigma and discrimination make women reluctant to seek care for TB until the disease is advanced. | Policies and programmes aimed at increasing women’s access to TB services should not only take these gender norms that disempower women into explicit consideration |
| Ochonma OG, Onuwjekwe OE. [55] | 2017 | Cross sectional study: willingness to pay study | 125 of 185 patients were registered with the TB treatment Unit of a tertiary hospital in Enugu as at the time of this study. | 80.0% of TB patients were willing to pay for their own use of TB treatment services while 62.4% of the patients were willing to make altruistic contributions for the very poor to benefit from TB services. | Most patients positively valued the provision of free TB services and were willing to pay for TB treatment for own use. The better-off ones were also willing to make altruistic contributions |
| Ukwaja KN, Alobu I, Gidado M, Onazi O, Oshi DC. [56] | 2017 | A prospective, non-randomised intervention study. | A total of 294 TB patients (respectively 173 and 121 in the control and intervention periods of 3months’ duration each) were registered in the study | Independent determinants of treatment success were female sex, human immunodeficiency virus negativity and receiving financial incentives | Financial incentives proved to be effective in improving treatment success and reducing loss to follow-up among poor TB patients in Nigeria |
| Ukwaja KN, Onyedum CC. [57] | 2013 | Commentary | Comment on article by Otu (2013) | Performance of TB service providers is limited by poor salaries and working condition, shortage of skilled TB workers, limited training opportunity and weak supervision | There is need for quality DOTS expansion. Addressing weaknesses in the health systems will enhance performance of the TB control programme in Nigeria |
| Oshi DC, Chukwu JN, Nwafor CC, Aguwa EN, Onyenoro UU, Meka A, et al. [58] | 2014 | A cross-sectional retrospective desk analysis using a questionnaire. | 280 patients' records of smear negative TB cases seen from April to June 2010 in six states of southern Nigeria | Only 3.6% had the second set of diagnostic tests as prescribed by the national guidelines. Only 2 patients (0.7%) were diagnosed in strict compliance with the national guidelines. | The adherence of health workers to the national guidelines for diagnosis of smear negative TB is apparently sub-optimal and needs improvement |
| Abdurrahman ST, Emenyonu N, Obasanya OJ, Lawson L, Dacombe R, Muhammad M, et al. [59] | 2014 | Prospective costing study | Costs related to installation of GeneXpert at five sites in Abuja were collected from receipts received from suppliers and normalized to USD 2012 values. | Installation varied widely between sites with sufficient space and power supply; sites with insufficient space or power supply and costs not directly associated with site installation | Space and power requirements have a significant effect on installation costs. |
| Obasanya J, Abdurrahman ST, Oladimeji O, Lawson L, Dacombe R, Chukwueme N, et al. [60] | 2015 | Secondary analysis of surveillance data | National TB control programme's routine surveillance database comprising data collected from 2008 to 2012 | The state case detection rate (CDR2012) in 2012could be predicted by the laboratory density. CDR2012 and laboratory density were correlated among states having < and > than 1 laboratory per 100 000 population. | There are large variations in laboratory density and case detection rate (CDR) across the Nigerian states. The CDR is associated with the laboratory density. |
| Onyeonoro UU, Chukwu JN, Nwafor CC, Meka AO, Omotowo BI, Madichie NO, et al. [61] | 2015 | Cross-sectional questionnaire survey. | 378 adult TB patients from 29 TB treatment centres in six states of Southern Nigeria selected using multi-stage sampling technique. | Patient characteristics and health systems factors like untidy environment, lack of basic service delivery infrastructure and staff attitude are associated with low patient satisfaction. | Patient- and health system–related factors were found to influence patient satisfaction and, hence, should be taken into consideration in TB service programming. |
| Tobin-West CI, Isodje A. [62] | 2016 | Mixed method: IDIs, Observation and cross-sectional questionnaire survey | Adult TB patients, health workers and TB treatment centres in four local government areas (2 urban and 2 rural) in Rivers State. | Health facilities lacked service delivery infrastructure, infection control measures and sufficient laboratory support for TB care. | Suboptimal quality of TB care in is characterised by limitations in health education and HCT of patients for HIV as well as laboratory support for TB care in rural health facilities |
| Onyenoro UU, Chukwu JN, Nwafor CC, Meka AO, Omotowo BI, Ogbudebe C, et al. [66] | 2015 | Mixed methods: cross-sectional questionnaire survey and patient exit interview. | 29 TB treatment facilities and 273 patients from six states in Southern Nigeria. 273 patients were interviewed to validate the information contained in the treatment cards. | Agreement between patient treatment card and facility TB register, and facility TB register and laboratory register were 97% and 85%, respectively. The lowest concordance was observed between the facility TB register and LGA TB register. | The study revealed that NTP data in Southern Nigeria is fairly reliable; however, there are variations observed among the states and at various levels. |
| Adamu AL, Gadanya MA, Abubakar IS, Jibo AM, Bello MM, Gajida AU, et al. [67] | 2017 | Cross sectional study: Retrospective review of patient records. | 1424 adult TB patients attending TB clinic of a teaching hospital in Kano State between January 2010 and December 2014. | Early deaths in this relatively young cohort is attributed to delay in diagnosis and treatment of TB, inadequate treatment of drug-resistant TB, and poor ART access. | Considerable expansion and improvement in quality of diagnosis and treatment services for TB and HIV are needed to achieve programme targets. |
| Ibrahim LM, Hadejia IS, Nguku P, Dankoli R, Waziri NE, Akhimien MO, et al. [68] | 2014 | Mixed methods study: Quantitative review of documents and qualitative study (IDIs and FGDs) | 378 patients' record reviewed. Adult TB patients selected through systematic random sampling | About 19% of TB patients interrupted their treatment, which was associated with living >5 km from TB treatment site, lack of knowledge of duration of treatment, cigarette smoking and unfriendly attitudes of health care workers. | Knowledge of the patients' duration of treatment, distance and health worker attitude were barriers to treatment adherence. |
| Okeibunor JC, Onyeneho GC, Chukwu JN, Post E. [69] | 2007 | Questionnaire | 12 randomly selected rural and urban Local Government Areas in southern Nigeria | TB patient had a median diagnostic-delay of 90 days. Delay in commencement of directly observed treatment short-course (DOTS) treatment was attributable to ignorance among patients and poor attitude of health workers. | Delay exists between recognition of symptoms and initiation of treatment in DOTS clinics partly because of ignorance among patients and poor health workers' attitude. |
| Ukwaja KN, Alobu I, Nweke CO, Onyenwe EC. [70] | 2013 | Cross-sectional study using standardised questionnaire | 450 adult TB patients enrolled in three rural (two mission/one public) hospitals in Ebonyi State. | The median total delay was 11 weeks, patient delay 8 and health system (HS) delay 3 weeks. Patient delay were predicted by age, distance and urban residence. Male gender and visit to non-NTP provider predicted HS delay. | TB treatment delays were high. Reduced delay may be achieved through improved access to care, further education of patients, engagement of informal care providers, and public-private partnerships. |
| Avong YK, Isaakidis P, Hinderaker SG, van der Bergh R, Obembe BO, Ekong E, et al. [71] | 2015 | Retrospective, observational cohort study, | All multi-drug resistant TB (MDR-TB) patients starting the intensive phase treatment at all MDR-TB treatment centres in Nigeria, from 1st February 2012 to 31st December 2013 | Gastro-intestinal, neurological, ototoxic and psychiatric were commonest adverse events (AEs). Ototoxic and psychiatric AEs were the most debilitating. There were significant inconsistencies in reporting adverse events. | MDR-TB Patients experienced a wide range of AEs. Early identification and prompt management as well as standardized reporting of AEs at all levels of healthcare is urgently needed. |
| Kuyinu YA, A. S. Mohammed AS, Adeyeye OO, Odugbemi BA, Goodman OO, Odusanya OO. [72] | 2016 | health facility survey questionnaire and observation of TB prevention practices and focus group discussions | 20 tuberculosis care facilities (16 public and 4 private) in Ikeja, Lagos. 40 health workers/FGD participants. | No clinics had a TB infection control plan. Weak managerial support, poor funding, under-staffing, lack of space and not wanting to be seen as stigmatizing against TB patients hindered TB infection control. | Tuberculosis infection control measures were not adequately implemented in health facilities in Ikeja, Nigeria. |
| Ogbonnaya LU, Chukwu JN, Uwakwe KA, Oyibo PG, Ndukwe CD. [73] | 2011 | Facility questionnaire survey, review of facility case notes and participant observation techniques | Twelve health facilities (4 per state per each of the 3 zones) from southern Nigeria | Implementation of the different aspects of the administrative control and work practice component of TB infection control measure range from 8.3% to 41.7% of the facilities. Though 66.7% of the consulting rooms were well ventilated, 25% of them were over crowded. | Urgent measures should be taken to reverse the poor infection control status of TB treatment centres. |
| WHO [1] | 2018 | Programme review | National TB Control Programme | Nigeria's performance indicators including domestic funding (8%); international funding (16%); and TB funding gap (76%) | Need to fill funding especially through domestic resource mobilisation. |
| Federal Ministry of Health (FMOH) [3] | 2014 | Situation analysis | National TB Control Programme | Insufficient budgetary allocation to TB and non-release of approved funds or delayed government funding; strategic plans and policy coordination framework for Tuberculosis (TB) control; stakeholders involved in strategic plans development and implementation; exclusion of TB from insurance benefit package; post and transfer of staff not transparent; community participation in TB care; TB is poorly integrated into primary health care and community; TB patients pay hidden fees for services. | A strategic plan for TB control is warranted. |
| FMOH [2] | 2013 | Prevalence study |  | Only 20% of active TB cases in Nigeria are notified. The TB prevalence rates in adults aged 15 years and above were estimated to be 318 per 100,000 population for smear-positive, and 524 for bacteriologically-conﬁrmed cases in Nigeria | Low case finding and notification despite high prevalence of TB cases. |
| FMOH [32] | 2008 | Programme review | National TB Control Programme | Programme description, annual achievements and challenges across thematic areas identified. | The weaknesses need to be addressed to improve the performance of the TB control programme. |
| Citro B, Mayowa J, Maher R, Shelbi S, Collen D, Odutose T, et al. [37] | 2018 | Descriptive, case study: document review and interviews | 36 stakeholders in the National TB Control Programme | Weak regulatory environment for PPM; Legal environment for TB control is weak/ absence of TB-specific legislation; TB patients pay hidden fees for service. | Need to establish the legal framework for TB control in Nigeria. |
| FMOH [31] | 2009 | Programme review | National TB Control Programme | Programme description, achievements and challenges in the preceding year across thematic areas identified. | The weaknesses need to be addressed to improve the performance of the TB control programme. |
| FMOH [63] | 2010 | Programme review | National TB Control Programme | Programme description, achievements and challenges in the preceding year across thematic areas identified. | The weaknesses need to be addressed to improve the performance of the TB control programme. |
| FMOH [64] | 2012 | Programme review | National TB Control Programme | Programme description, achievements and challenges in the preceding year across thematic areas identified. | The weaknesses need to be addressed to improve the performance of the TB control programme. |
| FMOH [65] | 2014 | Programme review | National TB Control Programme | Programme description, achievements and challenges in the preceding year across thematic areas identified. | The weaknesses need to be addressed to improve the performance of the TB control programme. |
| FMOH [30] | 2015 | Programme review | National TB Control Programme | Programme description, achievements and challenges in the preceding year across thematic areas identified. | The weaknesses need to be addressed to improve the performance of the TB control programme. |
| FMOH [44] | 2016 | Programme review | National TB Control Programme | Programme description, achievements and challenges in the preceding year across thematic areas identified. | The weaknesses need to be addressed to improve the performance of the TB control programme. |
